# Crosstalk between cancer cells and macrophages promotes OSCC cell migration and invasion through a CXCL1/EGF positive feedback loop

**DOI:** 10.1007/s12672-024-00972-8

**Published:** 2024-05-07

**Authors:** Wei Huang, Mingjing Jiang, Ying Lin, Ying Qi, Bo Li

**Affiliations:** 1 Experimental Teaching Center, School and Hospital of Stomatology, China Medical University, Liaoning Provincial Key Laboratory of Oral Diseases, Shenyang, 110001 China; 2 Department of Oral Anatomy and Physiology, Hospital of Stomatology, Jilin University, Jilin Provincial Key Laboratory of Oral Biomedical Engineering, Changchun, 130021 China

**Keywords:** OSCC, Macrophages, C-X-C motif chemokine ligand 1, Migration and invasion

## Abstract

**Background:**

C-X-C motif chemokine ligand 1 (CXCL1) and epithelial growth factor (EGF) are highly secreted by oral squamous cell carcinoma (OSCC) cells and tumor-associated macrophages, respectively. Recent studies have shown that there is intricate "cross-talk" between OSCC cells and macrophages. However, the underlying mechanisms are still poorly elucidated.

**Methods:**

The expression of CXCL1 was detected by immunohistochemistry in OSCC clinical samples. CXCL1 levels were evaluated by RT‒PCR and ELISA in an OSCC cell line and a normal epithelial cell line. The expression of EGF was determined by RT‒PCR and ELISA. The effect of EGF on the proliferation of OSCC cells was evaluated by CCK-8 and colony formation assays. The effect of EGF on the migration and invasion ability and epithelial-mesenchymal transition (EMT) of OSCC cells was determined by wound healing, Transwell, RT‒PCR, Western blot and immunofluorescence assays. The polarization of macrophages was evaluated by RT‒PCR and flow cytometry. Western blotting was used to study the molecular mechanism in OSCC.

**Results:**

The expression of C-X-C motif chemokine ligand 1 (CXCL1) was higher in the OSCC cell line (Cal27) than in immortalized human keratinocytes (Hacat cells). CXCL1 derived from Cal27 cells upregulates the expression of epithelial growth factor (EGF) in macrophages. Paracrine stimulation mediated by EGF further facilitates the epithelial-mesenchymal transition (EMT) of Cal27 cells and initiates the upregulation of CXCL1 in a positive feedback-manner. Mechanistically, EGF signaling-induced OSCC cell invasion and migration can be ascribed to the activation of NF-κB signaling mediated by the epithelial growth factor receptor (EGFR), as determined by western blotting.

**Conclusions:**

OSCC cell-derived CXCL1 can stimulate the M2 polarization of macrophages and the secretion of EGF. Moreover, EGF significantly activates NF-κB signaling and promotes the migration and invasion of OSCC cells in a paracrine manner. A positive feedback loop between OSCC cells and macrophages was formed, contributing to the promotion of OSCC progression.

**Supplementary Information:**

The online version contains supplementary material available at 10.1007/s12672-024-00972-8.

## Background

Oral squamous cell carcinoma (OSCC) is the most frequent type of head and neck squamous cell carcinoma (HNSCC). Despite the fact that multiple treatments are available for OSCC patients, the 5-year survival rate is less than 50% [1, 2]. The main causes of the poor prognosis are local invasion, recurrence, and lymph node metastasis of OSCC [3]. A thorough understanding of the underlying mechanisms of OSCC invasion and metastasis would facilitate the identification of new targets and therapies for OSCC patients.

The tumor microenvironment (TME) is a highly heterogeneous ecosystem that typically contains a collection of tumor cell populations, immune cells, tissue-specific resident cells and recruited stromal cells [4]. The development and progression of cancers are closely related to the tumor immune microenvironment (TIM). Tumor-associated macrophages (TAMs), the largest population of immune cells in the TIM, display two major phenotypes, M1 and M2 [5]. TAMs are predominantly the M2 macrophages, which promote cancer progression. In contrast, M1 macrophages exert an antitumor effect [6–8]. Recently, the link between TAMs and OSCC progression has also been uncovered [9]. Macrophages may represent potential therapeutic targets for OSCC.

At present, crosstalk between cancer cells and macrophages plays an important role in tumor progression. A previous study showed that hepatocellular progression was facilitated by crosstalk between macrophage-derived PGE and tumor UHRF1 [10]. HNSCC cells polarized monocytes into M2-like macrophages resulting in the promotion of HNSCC cell migration via the secretion of epithelial growth factor (EGF) [11]. In the OSCC microenvironment, OSCC cells secrete cytokines or chemokines to regulate the activities of macrophages [12, 13]. Infiltrated macrophages also interact with cancer cells to promote tumor progression in a paracrine manner [14, 15]. For example, TAMs promote OSCC cell invasion and metastasis via the secretion of CCL13 [16]. Moreover, macrophage-derived epithelial growth factor (EGF) induces OSCC migration and invasion via epithelial growth factor receptor (EGFR), and EGF has been linked with poor patient prognosis [17]. However, the underlying mechanism by which the crosstalk between macrophages and OSCC cells accelerates metastasis of OSCC remains unclear.

Tumor-derived CXCL1 plays an important role in migration and invasion by influencing the polarization of macrophages [18, 19]. Many previous studies have indicated that CXCL1 is highly expressed in the vast majority of OSCC cell lines and closely associated with OSCC invasion and metastasis [20–23], but the roles of tumor cell-secreted CXCL1 in the aggressiveness of OSCC have not been fully elucidated. Colorectal cancer cell-derived CXCL1 promotes the secretion of epithelial growth factor (EGF) in an autocrine manner by binding to CXCR2 in colorectal cancer cells [24]. CXCR2 has also been found in infiltrated macrophages in OSCC, prostate cancer, and hepatocellular carcinoma [25–27]. Whether OSCC cell-derived CXCL1 regulates the secretion of EGF via CXCR2 on the membrane of macrophages and further promotes OSCC progression needs to be determined.

It is crucial to find new therapeutic strategies and improve the clinical outcome of OSCC patients by completely understanding the molecular mechanism driving the interaction between tumor cells and macrophages. Our research demonstrated that OSCC cell-derived CXCL1 can stimulate the secretion of EGF via CXCR2 on the macrophage surface, and EGF significantly activates NF-κB signaling and promotes the migration and invasion of OSCC cells. Targeting CXCL1/CXCR2 or EGF/EGFR signaling might be a promising therapeutic approach to suppress OSCC progression and metastasis.

## Materials and methods

### Cell culture

The human oral tongue squamous carcinoma cell line Cal27, human epidermal keratinocyte cell line Hacat, and mouse acute monocytic leukemia cell line RAW264.7 were purchased from the China Center for Type Culture Collection. All of the cell lines were cultured in Dulbecco's modified Eagle's medium (VivaCell; Shanghai; China) with 10% fetal bovine serum (VivaCell; Shanghai; China), 100 U/ml penicillin, and 100 μg/ml streptomycin in a humidified 5% CO2 atmosphere at 37 °C.

### Reagents

Recombinant human CXCL1 and recombinant mouse EGF were purchased from R&D Systems Inc. (cat. no. 275-GR) and Abcam (cat. no. ab206643), respectively. AG1478 and SB225002 were purchased from Selleck Biotechnology Co., Ltd. (cat. no. S2728; cat. no. S7651). The antibodies used included rabbit anti-β-actin (cat. no. ab115777; Abcam), rabbit anti-E-cadherin (cat. no. ab40772 Abcam), rabbit anti-N-cadherin (cat. no. ab76011 Abcam), rabbit anti-CXCL1 (cat. no. ab206411 Abcam), rabbit anti-total EGFR (Zen Bio Science Co., Ltd), rabbit anti-phospho-EGFR (cat. no. R24173; Zen Bio Science Co., Ltd), rabbit anti-total P65 (cat. no. R25149; Zen Bio Science Co., Ltd), and rabbit anti-phospho-P65 (cat. no. 310013; Zen Bio Science Co., Ltd). DyLight 488 (A23220) and 800 (A23920) were purchased from Abbkine Scientific Co., Ltd. Anti-mouse PE CD86 (12-0862-81) and anti-mouse APC CD206 (17–2061-80) were purchased from Invitrogen, Thermo Fisher Scientific, Waltham, MA, USA. The anti-CXCL1 (YT2074) antibody for immunohistochemical staining was obtained from Immunoway, USA. The secondary antibody kit (GK600705) for IHC was purchased from Gene Tech, Shanghai, China.

### Tumor- and TAM-conditioned medium preparation

Cancer cell lines were cultured in complete medium, and the medium was replaced by serum-free medium when the cell density reached approximately 90%. After 48 h, the conditioned medium (CM) was harvested, centrifuged at 2000×*g* for 10 min, filtered through 0.22 mm filters, and stored at 80 °C. Regarding TAM-conditioned medium, Raw264.7 cells were stimulated with tumor-conditioned medium for 48 h, and then serum-free medium replaced the conditioned medium. After 24 h, the conditioned medium (CM) was harvested, centrifuged at 2000×*g* for 10 min, filtered through 0.22 mm filters, and stored at 80 °C. Tumor- or TAM-conditioned medium was defined as TCM or TAM-CM.

### Immunohistochemistry (IHC)

Five healthy tissues and ten OSCC tissues were obtained from the Department of Oral Pathology of China Medical University. Human participants were not included in the study. Both healthy tissues and OSCC tissues were obtained using the paraffin-embedded method. Immunostaining was performed using the avidin–biotin-peroxidase method. Sections were stained using primary anti-CXCL1 (1:100) antibody. Then, a secondary antibody kit was used. Finally, sections were stained with Mayer’s hematoxylin, and sections were observed using a light microscope (Nikon Eclipse Ts2R, Japan).

### Chemotactic migration and Matrigel invasion assays

For assessment of tumor cell migration, tumor cells (1 × 105) in 200 μl of serum-deprived medium were cultured in the upper chamber, and conditioned medium from macrophages stimulated with TCM or TCM and CXCL1 (100 ng/ml); TAM supernatant containing EGF (100 ng/ml) or EGF (100 ng/ml) and AG1478 (5 μM) with 10% FBS was added to the lower chamber. Moreover, cell invasion was assessed using a Transwell chamber (pore size 8 mm, Jet Biofil; Guangzhou; China) with Matrigel coating (BD Biosciences). For chemotactic migration and invasion, the non-migrated cells were removed from the surface of the upper chamber after 24 h, while after 48 h, the noninvasive cells were also scraped. The representative migrated or invaded cells were treated with 4% paraformaldehyde (product no. P0099; Beyotime Institute of Biotechnology) for 20 min and then with 0.5% crystal violet (product no. C0121; Beyotime Institute of Biotechnology) for 15 min. Cells were counted using a light microscope (Nikon Eclipse Ts2R, Japan) and analyzed by ImageJ software (National Institutes of Health, Bethesda, MD, USA).

### Scratch wound healing assay

Cell migration ability was assessed in vitro by wound healing. Cal27 cells (1 × 10^6/well) were seeded in 6-well plates treated with conditioned medium from macrophages stimulated with TCM or TCM and CXCL1 (100 ng/ml) or TAM-CM including EGF (100 ng/ml) or EGF and AG1478 (5 μM). After the tumor cells were grown to confluency, a scratch was created with a 200 µl pipette tip. Then, PBS was used to wash the plates several times. The plates were incubated for 24 h, and the tumor cells that migrated to heal the artificial wound were photographed with a light microscope (Nikon Eclipse Ts2R, Japan) and assessed with ImageJ software. The healing rate was calculated as (%) = (initial average scratch area − average scratch area at 24 h)/initial average scratch area × 100%.

### Real-time PCR (RT‒PCR)

RT‒PCR was used to measure the gene expression levels of CD206, CD86, Arg-1, EGF, CXCL1, cell transfection- and EMT-related markers in Raw264.7 cells and Cal27 cells. Total RNA was isolated by TRIzol Reagent (Takara Bio, Inc., Otsu, Japan), and extracted RNA was reversed transcribed by PrimeScript RT (Takara Bio, Inc., RR036A, Otsu, Japan). RT‒qPCR was performed using the SYBR Premix® Ex TaqTM Kit (Takara Bio, Inc, RR820A, Otsu, Japan) with the 7500 Fast System (Thermo Fisher Scientific). RT‒qPCR thermocycling conditions were as follows: 40 cycles of 95 °C for 30 s, 95 °C for 5 s, 60 °C for 34 s, 95 °C for 15 s, 60 °C for 60 s and 95 °C for 15 s. The internal control of the experiment was β-actin. Primers for β‑actin, CXCL1, EGF, E-cadherin, N-cadherin, Vimentin, CD206, CD86, and Arg-1 were obtained from Shanghai Sheng gong Biological Technology Co., Ltd. All primer sequences are shown in Table 1.

**Table 1:** Primers used for RT‒PCR

Gene	Forward primer 5′-3′	Reverse primer 5′-3′
E-Cadherin	CCTGGGACTCCACCTACAGAA	AGGAGTTGGGAAATGTGAGC
N-Cadherin	AACAGCAACGACGGGTTAGT	CAGACACGGTTGCAGTTGAC
Vimentin	AGGCGAGGAGAGCAGGATTT	AGTGGGTATCAACCAGAGGGA
CXCL1	AAGAACATCCAAAGTGTGAACG	CACTGTTCAGCATCTTTTCGAT
EGF	CATCATGGTGGTGGCTGTCTGC	CTCACACTTCCGCTTGGCTCAC
CD86	ACGGAGTCAATGAAGATTTCCT	GATTCGGCTTCTTGTGACATAC
CD206	CTCTGTTCAGCTATTGGACGC	CGGAATTTCTGGGATTCAGCTC
Arg-1	CATTGGCTTGCGAGACGTAGAC	GCTGAAGGTCTCTTCCATCAC
β-actin	GTGCTATGTTGCTCTAGACTTCG	ATGCCACAGGATTCCATACC

### Western blot analysis

Cal27 cells were washed with PBS and then lysed using RIPA buffer (product no. P0013B; Beyotime Institute of Biotechnology) including 1 mM PMSF (Product no. ST507; Beyotime Institute of Biotechnology) to extract total proteins. Next, the protein concentration was determined by a BCA protein assay kit (product no. P0012s; Beyotime Institute of Biotechnology). Total proteins were separated using 10% or 12% SDS‒PAGE, and electrophoresis was run at 150 V. The separated proteins were transferred to a PVDF membrane at 220 mA. The PVDF membrane was incubated with 5% nonfat dry milk at room temperature for 1 h to block the immunoblots and probed at 4 °C overnight with specific primary antibodies, including rabbit anti-β-actin (1:2000), rabbit anti-CXCL1 (1:1000), rabbit anti-E-cadherin (1:1000), rabbit anti-N-cadherin (1:1000), rabbit anti-total EGFR (1:1000), rabbit anti-phospho-EGFR (1:1000), rabbit anti-total P65 (1:1000), and rabbit anti-phospho-P65 (1:500). Then, the membranes were incubated with the secondary anti-rabbit antibody DyLight 800 goat anti-rabbit IgG (1:2000) at room temperature for 1 h. The gray values of the bands were analyzed by Odyssey CLX (LI-COR, Lincoln, NE, USA) and ImageJ software (National Institutes of Health, Bethesda, MD, USA).

### Enzyme-linked immunosorbent assay (ELISA)

Measurement of EGF and CXCL1 levels in the supernatant of the TAMs and Cal27 cells was conducted using mouse EGF ELISA kits (BOSTER Biological Technology Co. Ltd., Wuhan, China; EK0326) and human CXCL1 ELISA kits (R&D Systems Inc., Minneapolis, MN, USA; cat. no. #DY275), respectively. For the standard solution or sample, 100 μl/well was added to the 96-well containing specific antibody incubated at room temperature for 2 h. After washing three times with 300 μl diluted washing buffer, each well was incubated with the diluted HRP conjugate at room temperature for 1 h. Then, the diluted washing buffer was used to wash each well three times. Each well was incubated with 100 µl chromogenic substrate in the dark for 30 min. Finally, 90 μl stop solution was added to every well, and the absorbance of each well was measured by a microplate reader at 450 nm. By constructing a standard curve, the results of the sample solution were obtained.

### Immunofluorescence

The Cal27 cells were treated with 0.5% Triton solution for 10 min. PBS was used to wash the Cal27 cells three times. Then, the Cal27 cells were incubated with primary antibodies against one of the EMT markers (E-cadherin) at 4 °C overnight. The cells were washed three times with PBS again. The cells were incubated with the corresponding secondary anti-rabbit antibodies with DyLight 488 (1:200) at room temperature for 1 h. Finally, the cells were counterstained with DAPI at room temperature for 10 min. Moreover, tissue sections were incubated using primary anti-CXCL1 (1:100) antibody. Then, a secondary antibody kit was used. The staining was observed and photographed with a fluorescence microscope (Nikon Eclipse Ts2R, Japan).

### Cell proliferation assay

The rate of cell proliferation was determined using a Cell Counting Kit-8 assay (product no. C0038; Beyotime Institute of Biotechnology) according to the manufacturer’s instructions. Cal27 cells were plated in 96-well plates at a ratio of 3 × 10^3/well. Then, the cells were incubated in TAM-CM with EGF (100 ng/ml) or EGF (100 ng/ml) and AG1478 (5 μM) for 24 and 48 h. Ten microliters of CCK-8 solution was added to every well, and the cells were cultured at 37 °C for 1 h. The absorbance of each well was measured at 450 nm (Tecan Mechelen, Belgium).

### Colony formation assay

Cal27 cells were cultured in 6-well plates at a ratio of 1 × 10^3/well and stimulated with TAM-CM containing EGF (100 ng/ml) or EGF (100 ng/ml) and AG1478 (5 μM) for 2 weeks. The incubation medium was replaced every two days. Subsequently, the cells were treated with 4% paraformaldehyde and then with 0.5% crystal violet for 30 min, and colonies were counted with a light microscope.

### Transfection of Cal27 cells

Cal27 cells (1 × 10^5/well) were seeded in 6-well plates and transfected with double-stranded small interfering RNA (siRNA) (Shanghai Gene Pharma Co., Ltd., Shanghai, China). Sense and antisense strands for siRNAs were as follows: si-CXCL1-1 (5′-ACUCAAGAAUGGGCGGAAATT-3′) (5′-UUUCCGCCCAUUCUUGAGUTT-3′); si-CXCL1-2 (5′-CCAAGAACAUCAAAGUGUTT-3′) (5′-ACACUUUGGAUGUUCUUGGTT-3′); and si-CXCL1-3 (5′-GAUGCUGAACAGUGACAAATT-3′) (5′-UUUGUCACUGUUCAGCAUCTT-3′). Cal27 cells transfected for 24 h were used for subsequent experiments.

### Flow cytometry

After RAW264.7 cells were cultured with TCM or TCM and CXCL1 for 48 h, they were stained with anti-mouse PE CD86 (Thermo Fisher Scientific, Waltham, MA, USA) and anti-mouse APC CD206 (Thermo Fisher Scientific, Waltham, MA, USA) on ice for 20 min according to the dilution ratio recommended by the manufacturers. Data analysis was performed using FlowJo software.

### Statistical analysis

Prism 8.0 for Windows (GraphPad Software, Inc., La Jolla, CA, USA) was used for data analysis. Measurement data are presented as the mean ± standard deviation (SD), and statistical significance between groups was determined by Student’s t test or analysis of variance (ANOVA). Western blotting, wound healing assay and Transwell assay results were assessed by ImageJ software. Differences were considered statistically significant at *P < 0.05, **P < 0.01, and ***P < 0.001.

## Results

### CXCL1 is highly expressed in OSCC tissues.

**Fig. 1:**
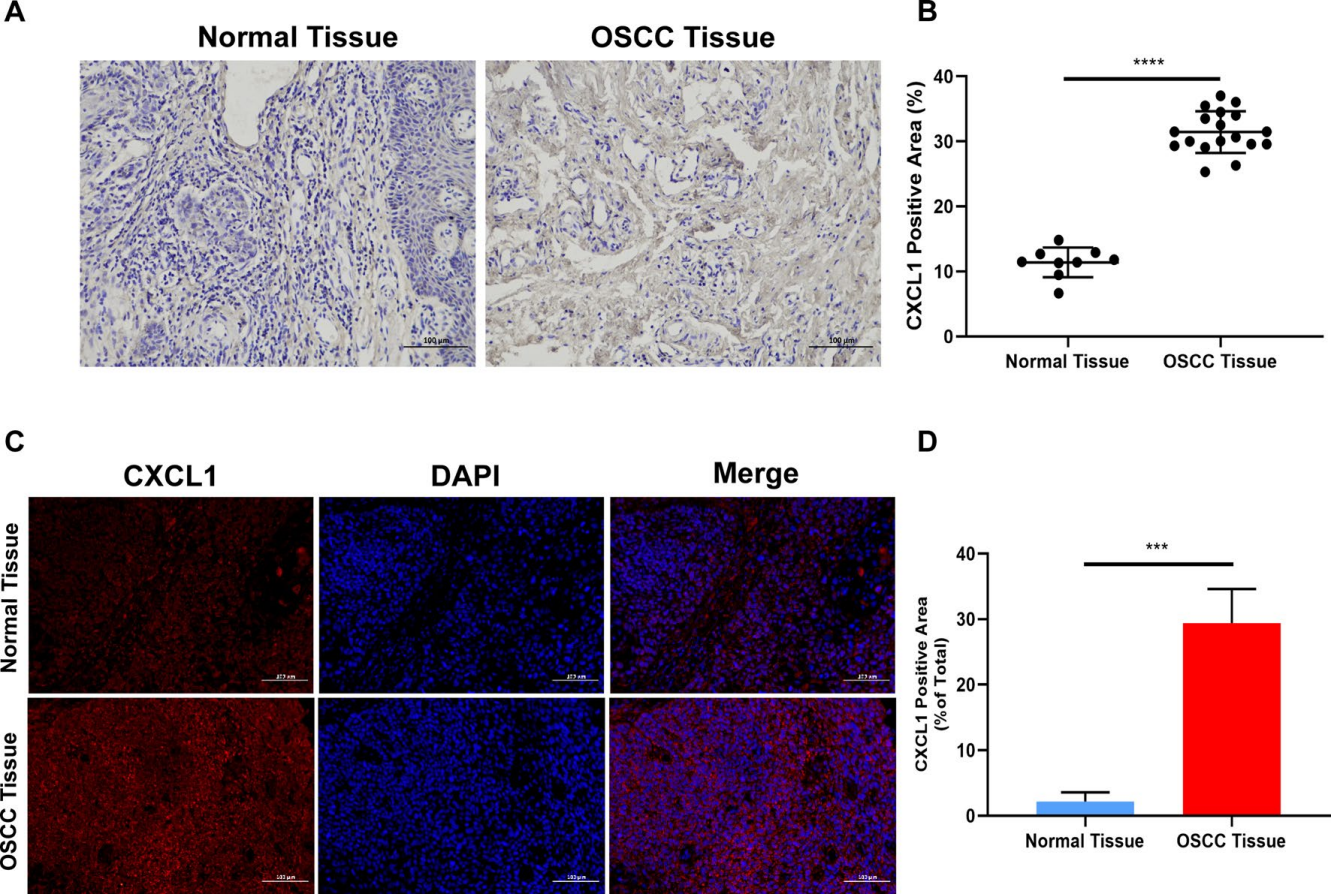
**A** Representative images of CXCL1 staining in healthy tissues and OSCC tissues. Color of nucleus (blue) and CXCL1 (brown). **B** Semi-quantitative analysis of IHC staining. **C** Detection of CXCL1 expression by immunofluorescence staining in healthy tissues and OSCC tissues. Color of nucleus (blue) and CXCL1 (red). **D** Semi-quantitative analysis of immunofluorescence staining

A previous study demonstrated that CXCL1 was highly expressed in OSCC tissues [20]. In our study, CXCL1 expression was determined in healthy tissues and OSCC tissues by immunohistochemical staining and immunofluorescence, as shown in Fig. 1A–D. The results demonstrated that CXCL1 was overexpressed in OSCC tissues. High levels of CXCL1 in OSCC have been related to the malignant progression of OSCC. The role of CXCL1 in OSCC and the underlying mechanism need to be studied.

### CXCL1 promotes OSCC cell migration and invasion through crosstalk with macrophages

**Fig. 2:**
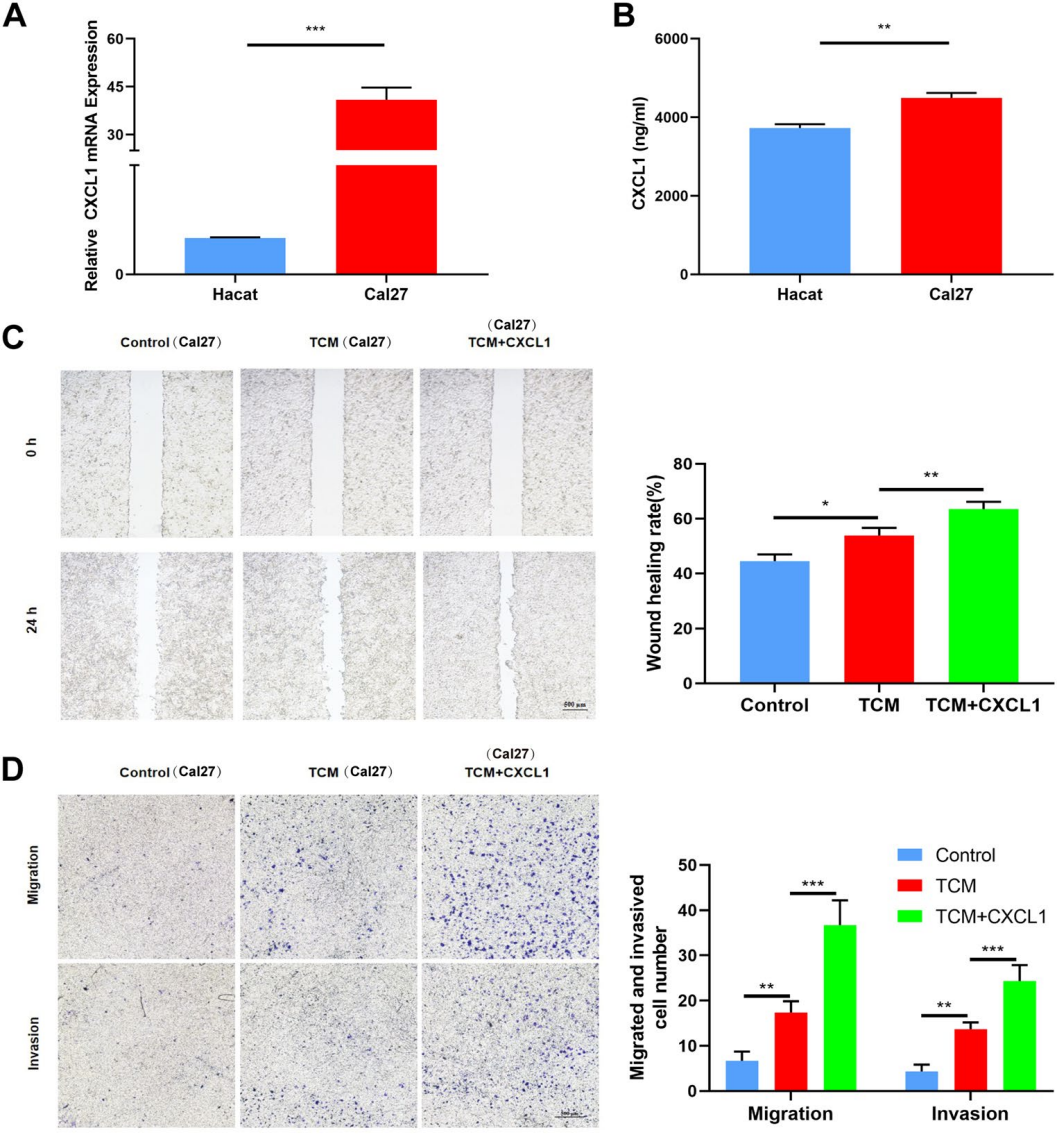
CXCL1 induces OSCC cell migration and invasion via macrophage crosstalk. **A** Measurement of CXCL1 mRNA expression by RT‒PCR in Hacat cells and Cal27 cells. **B** Secretion levels of CXCL1 in Hacat cells and Cal27 cells were measured by ELISA. **C** Wound healing assays showed the migration of Cal27 cells. **D** Transwell assays showed the migratory and invasive abilities of Cal27 cells. Bar graphs are presented as the mean ± SD (n = 3). *P < 0.05, **P < 0.01, ***P < 0.001 vs. the control

In addition to OSCC tissues, CXCL1 is also highly expressed in many OSCC cell lines. Our PCR and ELISA results consistently indicated that the mRNA level and secretion level of CXCL1 were higher in Cal27 cells than in Hacat cells (Fig. 2A, B). However, the roles of tumor cell-secreted CXCL1 in the progression of OSCC are not fully clear. It has been reported that the CXCL1-mediated interaction of cancer cells with tumor-associated macrophages promotes tumor progression in bladder cancer. To verify that CXCL1 promotes OSCC cell migration and invasion through crosstalk with macrophages, Raw264.7 cells were cultured with tumor conditional medium (TCM) or TCM and CXCL1. Then, the conditioned medium of macrophages stimulated with TCM or TCM and CXCL1 was used to stimulate Cal27 cells. The wound-healing and Transwell assays demonstrated that the supernatant of TCM-induced macrophages promoted Cal27 cell migration and invasion. Moreover, the supernatant of macrophages treated with TCM and CXCL1 further induced migration and invasion (Fig. 2C, D). These results illustrated that CXCL1 may induce the migration and invasion of OSCC cells through crosstalk with macrophages.

### CXCL1 promotes M2 macrophage polarization and upregulates macrophage EGF

**Fig. 3:**
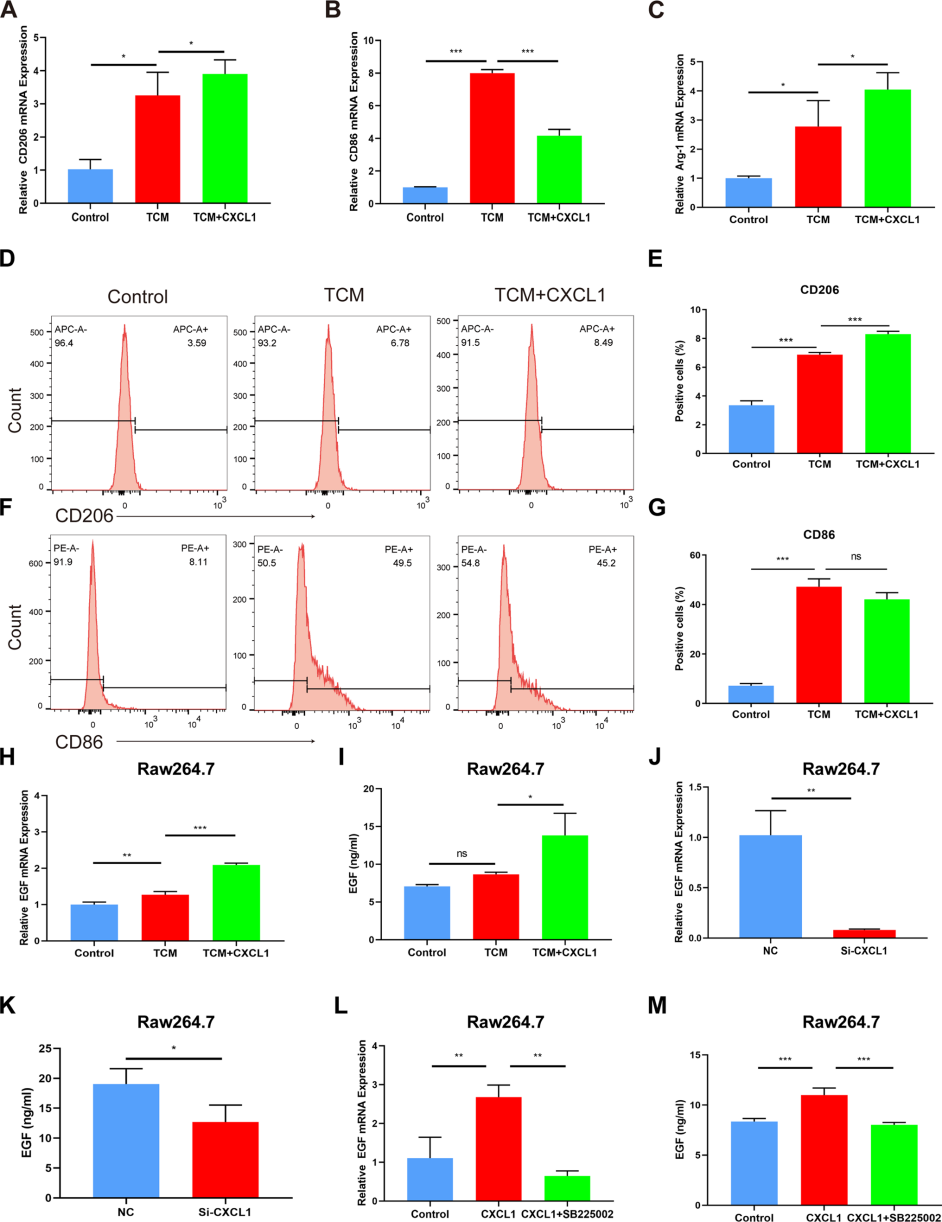
M2 macrophage polarization is enhanced and EGF expression is upregulated in macrophages. **A**–**C** Gene expression of CD206, CD86, and Arg-1 was measured by RT‒PCR. **D**–**G** Flow cytometric analysis of surface markers of CD206 and CD86 in RAW264.7 cells incubated with CXCL1 (100 ng/ml) or without CXCL1-containing tumor-conditioned medium. **H**, **I** mRNA and protein expression levels of EGF in RAW264.7 cells incubated with or without CXCL1 (100 ng/ml) in tumor-conditioned medium. **J**, **K** mRNA and protein expression of EGF in RAW264.7 cells incubated with Cal27-transfected supernatant or Cal27-CM. **L** RT‒PCR was used to examine the mRNA expression of EGF in RAW264.7 cells. **M** Immunosorbent assay (ELISA) showing the protein level of EGF in RAW264.7 cells. Bar graphs are presented as the mean ± SD (n = 3). *P < 0.05, **P < 0.01, ***P < 0.001 vs. the control

According to the results above, CXCL1 could induce OSCC cell migration and invasion via macrophages. Thus, we further determined whether CXCL1 affects the polarization of macrophages to promote the aggressive behavior of OSCC. The results demonstrated that recombinant CXCL1 increased the mRNA levels of Arg-1 and CD206 while decreasing the level of CD86 in macrophages (Fig. 3A–C). We also found that compared to the control group, the group treated with recombinant CXCL1 exhibited upregulated levels of CD206 (M2 surface marker) and reduced levels of CD86 (M1 surface marker) (Fig. 3D–G). It has been reported that colorectal cancer cell-derived CXCL1 promotes the secretion of epithelial growth factor (EGF) in an autocrine manner by binding to C-X-C motif chemokine receptor 2 (CXCR2) in colorectal cancer cells [28]. Moreover, CXCR2 was also expressed on the macrophage surface and found to mediate tumor invasion and metastasis [25], so we hypothesized that OSCC cell-derived CXCL1 may promote macrophages to secrete EGF via CXCR2. Compared with TCM, TCM combined with recombinant CXCL1 further induced the secretion of EGF in macrophages (Fig. 3H, I). However, knockdown of CXCL1 in Cal27 cells led to the opposite effect (Fig. 3J, K). We used an inhibitor of CXCR2 (SB225002) to block the CXCL1/CXCR2 pathway [29]. The gene and protein levels of EGF were reversed (Fig. 3L, M). These results demonstrated that CXCL1 promoted M2 macrophage polarization and induced the secretion of EGF in macrophages.

### EGF promotes OSCC cell proliferation, migration and invasion

**Fig. 4:**
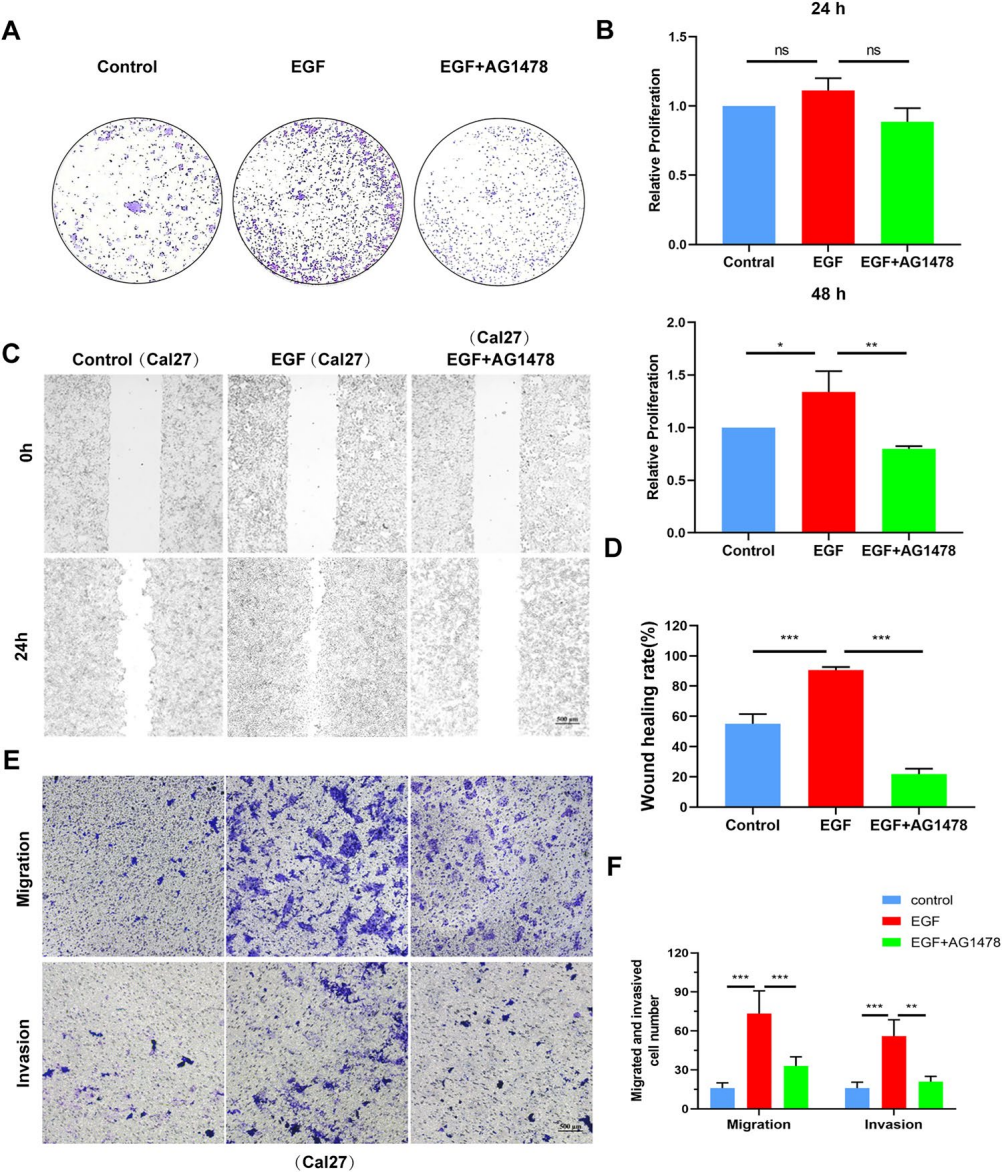
EGF induces the proliferation, migration and invasion of OSCC cells. **A** Colony formation assay of Cal27 cells stimulated with TAM-CM containing EGF or EGF and AG1478 for 2 weeks. **B** Proliferation assay of Cal27 cells cultured with TAM-CM containing EGF or EGF and AG1478 for 24 h or 48 h. **C**, **D** Wound healing assays determined the migration of Cal27 cells. **E**, **F** Transwell assays determined the migratory and invasive abilities of Cal27 cells. Bar graphs are presented as the mean ± SD (n = 3). *P < 0.05, **P < 0.01, ***P < 0.001 vs. the control

In the tumor environment, autocrine EGF has multiple functions, such as promoting the invasion, metastasis and EMT of cancer cells [6, 30]. In previous research, the functions of EGF mainly depended on its receptor EGFR [31]. To further explore whether macrophage-derived EGF exerts a cancer-promoting effect via EGFR, recombinant-incubated Cal27 cells were treated with or without an inhibitor of EGFR (AG1478). Then, colony formation and CCK-8 assays were used to evaluate cell proliferation and viability. The colony formation results revealed that recombinant EGF significantly increased the proliferation ability of Cal27 cells, while AG1478 treatment led to a decrease in cell viability (Fig. 4A). There was no difference in the proliferation ability of Cal27 cells at 24 h. However, after stimulation with recombinant EGF for 48 h, the proliferation ability of Cal27 cells was increased, and these effects could be abrogated by AG1478 treatment (Fig. 4B). The 24 h wound-healing assay was employed to determine the migration ability of the Cal27 cells. The increased healing rate of Cal27 cells induced by recombinant EGF was decreased by AG1478 (Fig. 4C, D). Moreover, the results of the Transwell assay showed that the migration and invasion abilities of Cal27 cells were prominently increased following 24 h of EGF treatment. We also performed knockdown experiments by using an inhibitor of EGFR, AG1478. The results showed that AG1478 could abrogate the increased cell migration and invasion (Fig. 4E, F). In summary, these data demonstrated that EGF promoted the proliferation, migration and invasion of OSCC cells.

### EGF regulates OSCC cell EMT

**Fig. 5:**
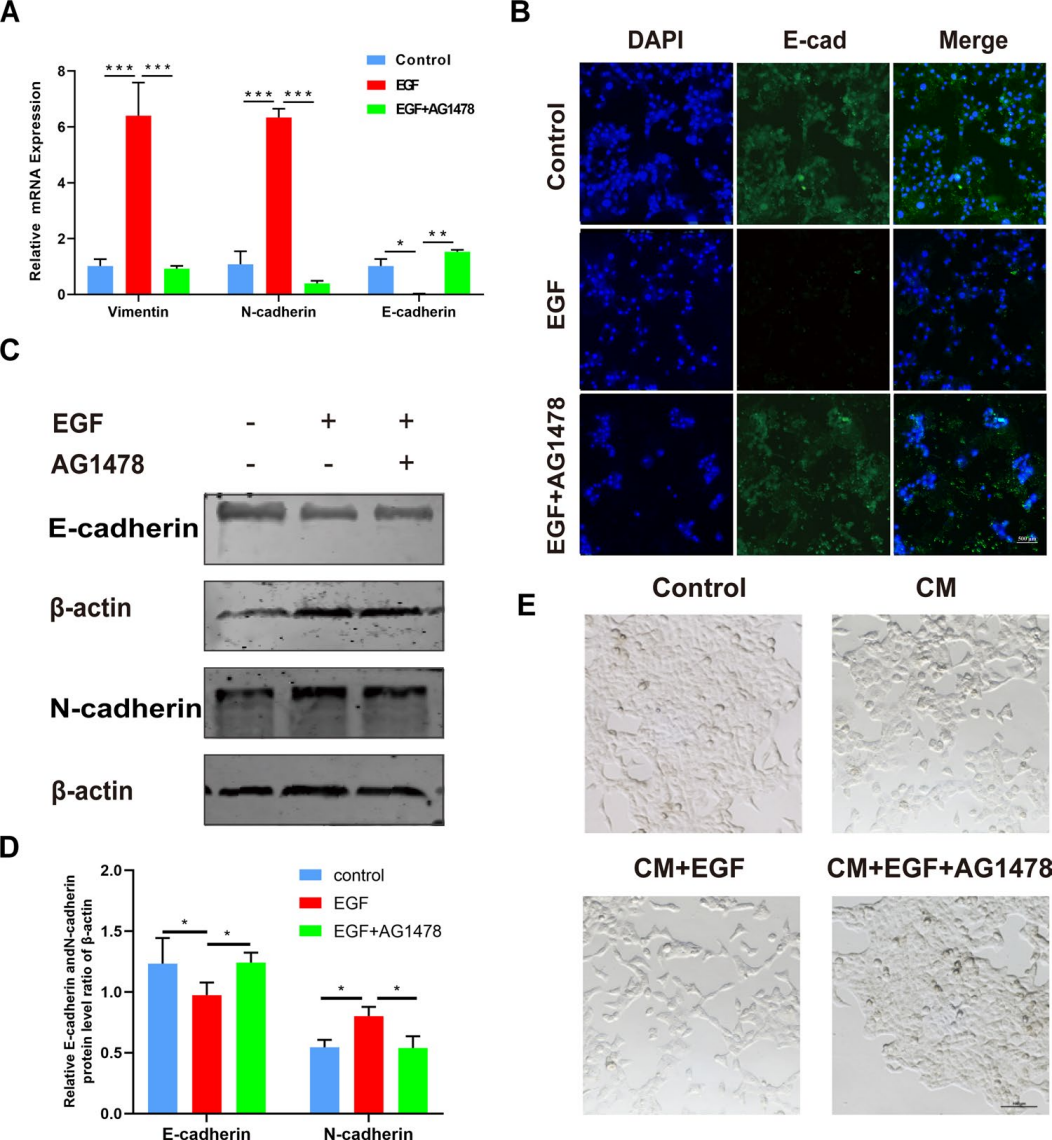
EGF induces EMT in OSCC cells. **A** Measurement of E-cadherin, N-cadherin and Vimentin mRNA expression by RT‒PCR. **B** Measurement of E-cadherin expression by immunofluorescence staining in Cal27 cells. **C**, **D** Western blot analysis of the expression of E-cadherin and N-cadherin in Cal27 cells treated with EGF or EGF and AG1478-containing TAM-CM. β-Actin was used as an internal control. **E** Cal27 cells were treated with EGF or EGF and AG1478-containing TAM-CM. Morphological changes in the indicated Cal27 cells were evaluated using inverted fluorescence microscopy. Bar graphs are presented as the mean ± SD (n = 3). *P < 0.05, **P < 0.01, ***P < 0.001 vs. the control

Epithelial-mesenchymal transition (EMT) is an important underlying mechanism of primary tumorigenesis and metastasis [31]. During EMT, epithelial cells transform from epithelial cells highly expressing epithelial markers (E-cadherin) to mesenchymal cells overexpressing mesenchymal markers (N-cadherin and Vimentin), which then promote the migration and invasion of cancer cells [32]. Subsequently, we further investigated whether EGF enhanced the migration and invasion of Cal27 cells by inducing EMT. TAM-CM supplemented with EGF or EGF and AG1478 was used to incubate Cal27 cells for 48 h. The RT‒PCR results showed that EGF increased the mRNA levels of Vimentin and N-cadherin and decreased the mRNA level of E-cadherin in Cal27 cells. However, AG1478 had exactly the opposite effect (Fig. 5A). Western blot analysis and immunofluorescence staining showed that the EGF-induced suppression of E-cadherin was abrogated by AG1478. Simultaneously, the EGF-induced high protein level of N-cadherin was decreased by AG1478 treatment (Fig. 5B–D). As shown in Fig. 5E, the morphology of Cal27 cells became elongated and spindle-shaped when cells were stimulated by EGF, while the Cal27 cells became cobblestone-shaped when they were treated with AG1478. The above results revealed that EGF could greatly promote the EMT of OSCC cells.

### EGF induces OSCC invasion and metastasis through the EGFR/NF-κB signaling pathway

**Fig. 6:**
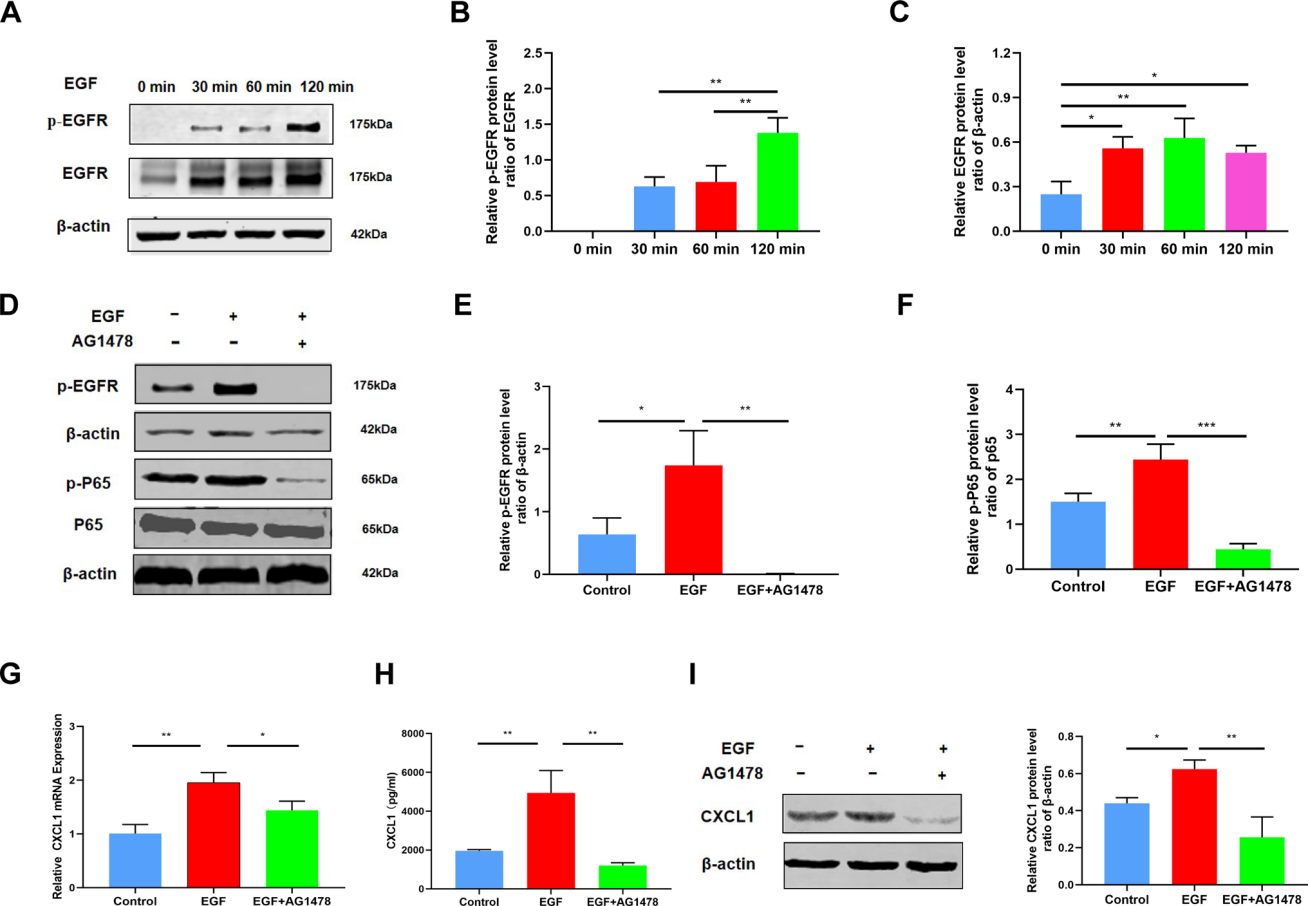
EGF promotes OSCC progression by activating the EGFR/NF-κB pathway. **A** Cal27 cells were treated with EGF (100 ng/ml) for 0, 30, 60, and 120 min, and the expression of EGFR was detected by Western blot analysis. **B**, **C** Quantified detection of the EGFR expression. **D** Western blot analysis of the expression of EGFR and members of the NF-κB signaling pathway in Cal27 cells treated with EGF or EGF and AG1478. **E**, **F** Quantitative analysis of Western blots in (**D**) Cal27 cells. **G** mRNA expression of CXCL1 in Cal27 cells treated with EGF (100 ng/ml) or EGF and AG1478 (5 μM) in TAM-CM. **H** Measurement of CXCL1 levels in treated Cal27 cells by ELISA. **I** Western blot analysis of the expression of CXCL1 in Cal27 cells treated with EGF or EGF and AG1478-containing TAM-CM. Bar graphs are presented as the mean ± SD (n = 3). *P < 0.05, **P < 0.01, ***P < 0.001 vs. the control

Former studies have revealed that a number of signaling pathways activated by EGF could result in increased cell migration and invasion [5, 27]. EGF mainly functions by binding to EGFR. Therefore, an inhibitor of EGFR (AG1478) was used to suppress EGF/EGFR signaling [33]. Western blot analysis demonstrated that EGFR expression was slightly upregulated and that the p-EGFR signaling pathway was significantly activated in Cal27 cells stimulated with recombinant EGF (100 ng/ml) for 30, 60 and 120 min (Fig. 6A–C). Furthermore, Western blot analysis was used to detect the protein expression of NF-κB pathway members in Cal27 cells treated with EGF (100 ng/ml) or EGF (100 ng/ml) combined with AG1478 (5 μM). The analysis showed that after treatment with EGF (100 ng/ml), phosphorylation of EGFR and p65 in Cal27 cells was increased. However, AG1478 inhibited the activation of the NF-κB/p-65 pathway (Fig. 6D–F). A recent study identified the NF-κB/p-65 pathway as the mediator of OSCC cell migration and invasion [34–36]. Therefore, these results demonstrated that EGF may promote the invasion and migration of OSCC cells through the EGFR/NF-κB signaling.

Previous studies have revealed that EGF increases the production of CXCL1 in hepatocellular carcinoma cells [37]. Therefore, it was extremely necessary to determine whether CXCL1 and EGF could mediate crosstalk between OSCC cells and macrophages and further induce the aggressiveness of OSCC cells in the tumor microenvironment. As shown in Fig. 6G–I, the mRNA expression of CXCL1 was upregulated in Cal27 cells after stimulation with recombinant EGF. Moreover, the protein expression of EGF in Cal27 cells treated with EGF was also upregulated. However, when we used an inhibitor of EGFR (AG1478) to inhibit the EGF/EGFR signaling pathway, the level of CXCL1 was also suppressed [30]. These results indicated that CXCL1 could promote OSCC cell invasion and metastasis through a CXCL1/EGF-mediated positive feedback loop in macrophages and OSCC cells.

## Discussion

The OSCC microenvironment is a chronic inflammatory environment. Numerous previous studies have revealed that the poor prognosis of OSCC patients is related to macrophage infiltration [38]. In the OSCC microenvironment, cytokines and chemokines have a crucial effect on tumor cells, and they also mediate the crosstalk between tumor cells and infiltrated macrophages. Here, we made efforts to investigate the interaction between tumor cells and macrophages, as well as the underlying mechanism by which macrophages contribute to the invasion and metastasis of OSCC cells. This study may facilitate the development of novel therapeutic approaches for OSCC.

Previous studies indicated that the gene and protein expression levels of CXCL1 in OSCC cells were much higher than those in normal epithelium cells [20]. In our current investigation, we found heightened expression of CXCL1 in OSCC tissues and OSCC cells. Moreover, CXCL1 promoted OSCC cell migration and invasion via macrophages. Tumor-derived CXCL1 has been reported to promote malignant behavior by polarizing macrophages [18, 38]. Subsequently, the phenotype of macrophages was evaluated and the results were consistent with the above research showing that macrophages stimulated by CXCL1 underwent polarization into the M2 phenotype and secreted high levels of EGF.

Persistent chronic inflammation in cancer tissue gives rise to inhibition of antitumor immunity via a variety of mechanisms. For example, macrophages infiltrating the tumor microenvironment are polarized into activated (M1) and alternatively activated (M2) phenotypes, and anti-inflammatory and immunosuppressive M2 TAMs can induce tumor invasion and metastasis [39, 40]. Whether CXCL1-induced EGF in M2 macrophages regulates OSCC cell proliferation, migration and invasion, as well as EMT in Cal27 cells was further examined in our research. We observed that cell proliferation, migration and invasion were enhanced by recombinant EGF. Meanwhile, the epithelial marker E-cadherin was downregulated, while the mesenchymal marker N-cadherin was upregulated in Cal27 cells. These results indicated that M2 macrophage-derived EGF play an important role in the invasive behavior of OSCC.

Macrophages secreting EGF could induce the invasion and metastasis of tumor cells via the EGF/EGFR signaling pathway [41]. The downstream signaling of EGF/EGFR that mediates the invasion and migration of OSCC cells is unclear. Metastasis of OSCC was controlled by multiple molecular mechanisms, such as p65 phosphorylation and activation of other intracellular signaling pathways [42, 43]. In our research, we noticed that the levels of p-EGFR and p65 were significantly activated after stimulation with recombinant EGF. Moreover, the phosphorylation level of p65 was prominently reversed by an inhibitor of EGFR (AG1478). The NF-κB/p-65 pathway has been reported to be a mediator of OSCC metastasis [34–36]. These results indicated that EGF may induce the migration and invasion of OSCC cells through the EGFR/NF-κB signaling pathway.

The crosstalk between tumor cells and macrophages during malignant tumor progression has been increasingly reported [39, 44, 45]. Our data demonstrated that CXCL1 could induce OSCC migration and invasion via inducing the secretion of EGF in macrophages. In addition, the expression of CXCL1 in Cal27 cells was upregulated after stimulation with recombinant EGF, and blockade of the EGF/EGFR axis reduced the secretion of CXCL1, which indicated that a CXCL1/EGF positive feedback loop was formed between OSCC cells and macrophages.

## Conclusions

**Fig. 7:**
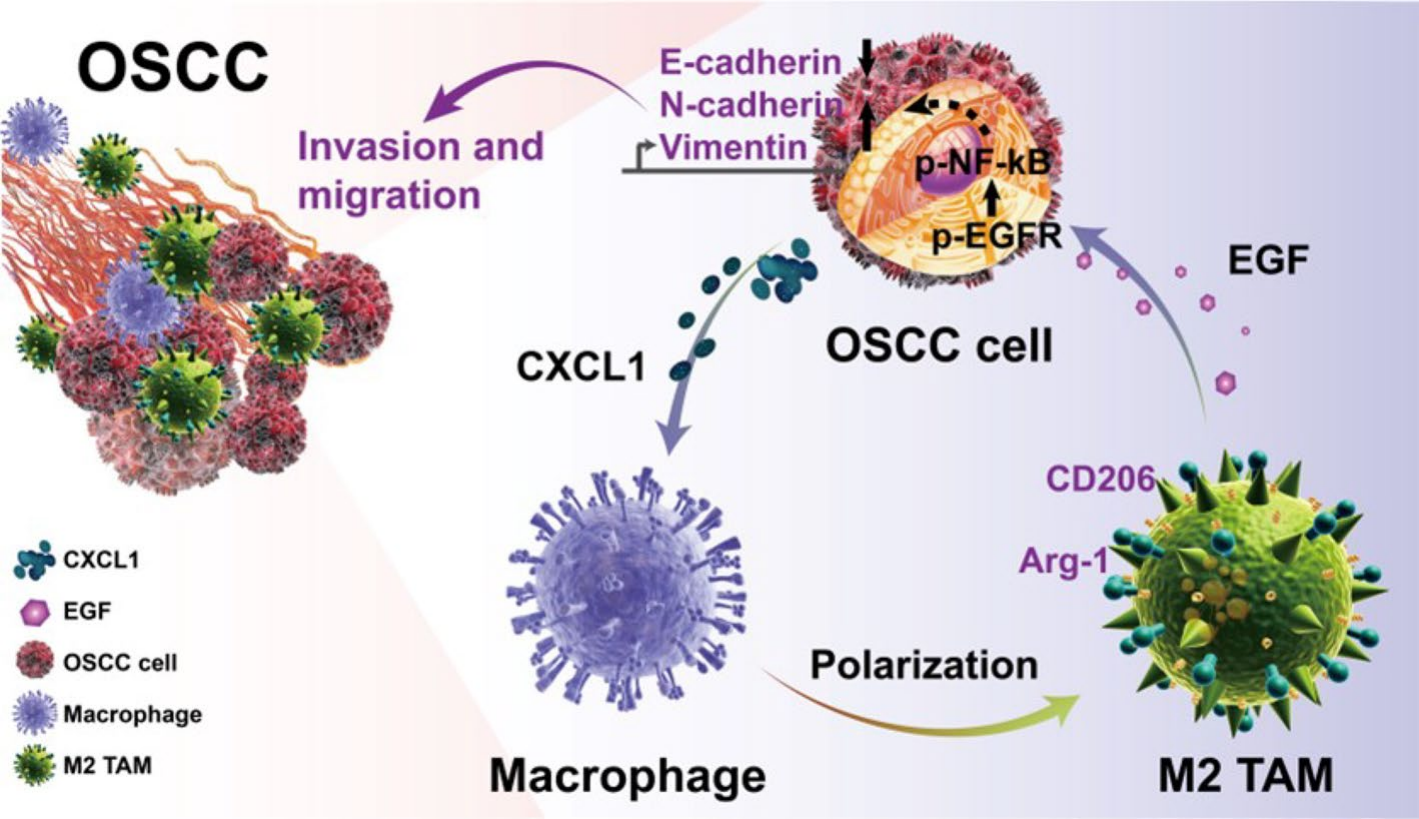
Model of crosstalk between cancer cells and activated macrophages promoting OSCC progression via the CXCL1/EGF feedback loop. Tumor-derived CXCL1 facilitates the secretion of EGF in TAMs, while TAM-derived EGF induces the activation of EGFR/NF-κB in OSCC cells, which accelerates tumor cell migration and invasion

The present study provides evidence that CXCL1 secreted by cancer cells stimulates macrophages to secrete EGF, which contributes to tumor cell invasion and metastasis via EGFR in OSCC cells. The EGF-induced malignant behavior of OSCC may be attributed to the activation of EGFR/NF-κB in Cal27 cells (Fig. 7). These results not only elucidate how tumor-derived chemokines drive the polarization of macrophages but also provide unique insight into how polarized macrophages contribute to cancer invasion and metastasis. Overall, our study revealed a positive feedback loop between macrophages and OSCC cells, and targeting CXCL1/EGF/NF-κB signaling may be a novel strategy for OSCC treatment.

### Supplementary Information

Below is the link to the electronic supplementary material

Supplementary file1 (DOCX 7507 KB)

## Data Availability

All data generated or analyzed during this study are included in this published article.

## Abbreviations

HNSCC: Head and neck squamous cell carcinoma
OSCC: Oral squamous cell carcinoma
TME: Tumor microenvironment
TIM: Tumor immune microenvironment
CXCL1: C-X-C motif chemokine ligand 1
EGF: Epithelial growth factor
EGFR: Epithelial growth factor receptor
CXCR2: C-X-C motif chemokine receptor 2
EMT: Epithelial-mesenchymal transition
IHC: Immunohistochemistry
