# Supplementary material for: Crosstalk between cancer cells and macrophages promotes OSCC cell migration and invasion through a CXCL1/EGF positive feedback loop
Source: Discov Oncol. 2024 May 7;15:145. doi: 10.1007/s12672-024-00972-8 (PMC11076430; doi:10.1007/s12672-024-00972-8)
Supplement: Supplementary file 1 — Supplementary file1 (DOCX 7507 KB) [file 12672_2024_972_MOESM1_ESM.docx]

Figure 5C (E-cadherin)


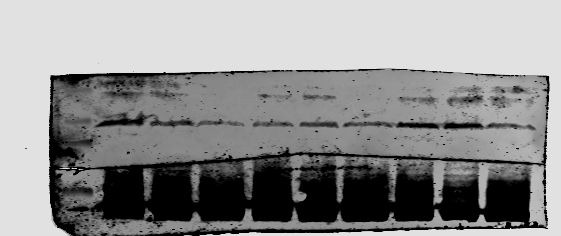


β-actin

40 KD


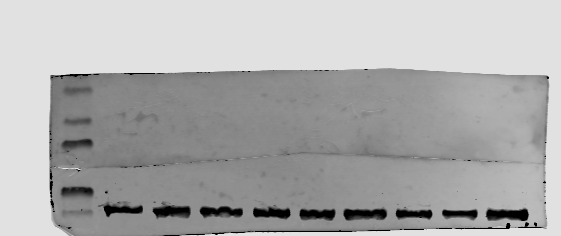


E-cadherin

70 KD

100 KD

35 KD

40 KD

55 KD


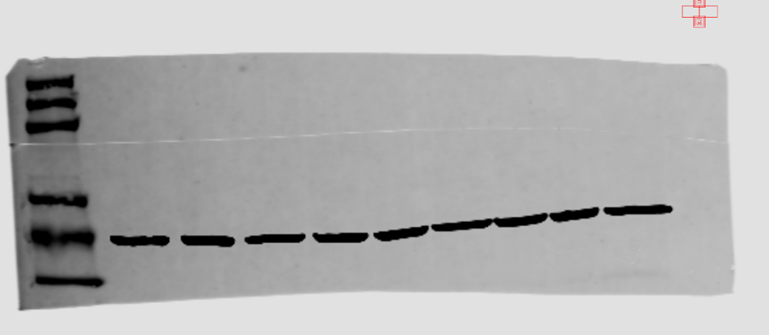


β-actin

130 KD

35 KD

40 KD

55 KD

70 KD

100 KD


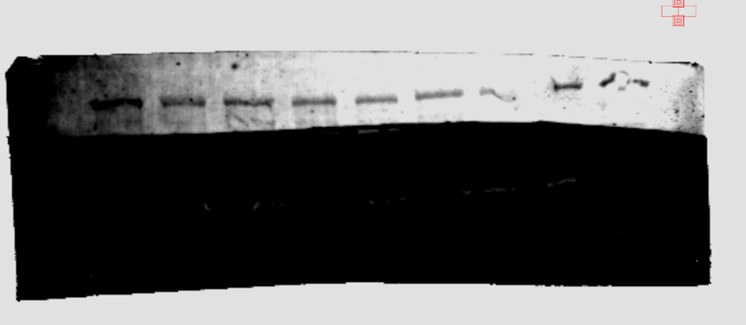


E-cadherin

100 KD

N-cadherin


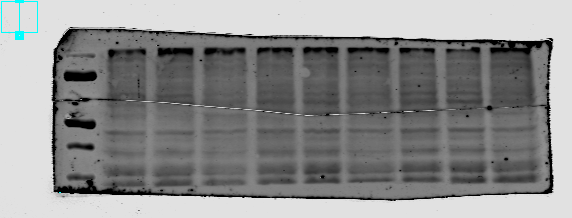


N-cadherin

100KD

70 KD

55 KD

40 KD

35 KD


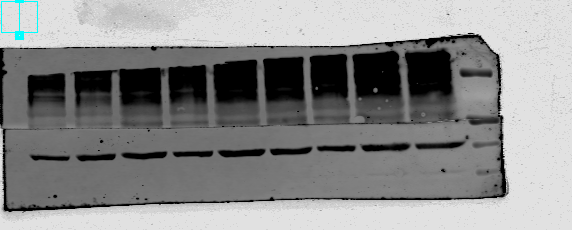


β-actin

70 KD

35 KD

55 KD

40 KD


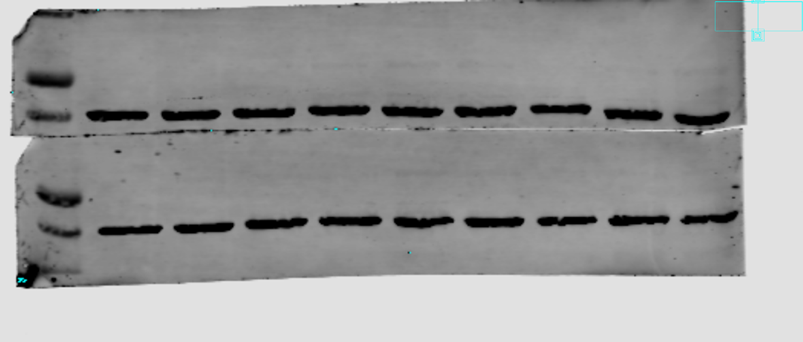


β-actin

40 KD

40 KD

35 KD

35 KD

55 KD

130 KD

100 KD

70KD

170 KD

130 KD

100 KD

170 KD


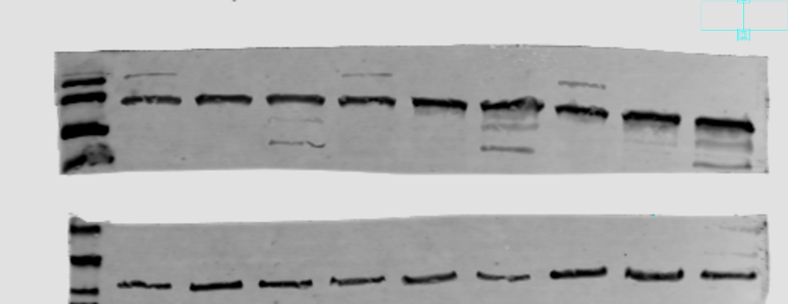


N-cadherin

Figure 6I (CXCL1)

55 KD


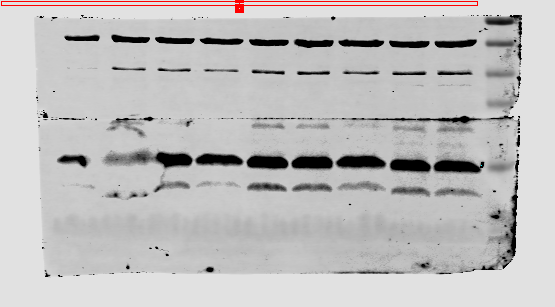


β-actin

25 KD

35 KD

40 KD

15 KD

10 KD

CXCL1


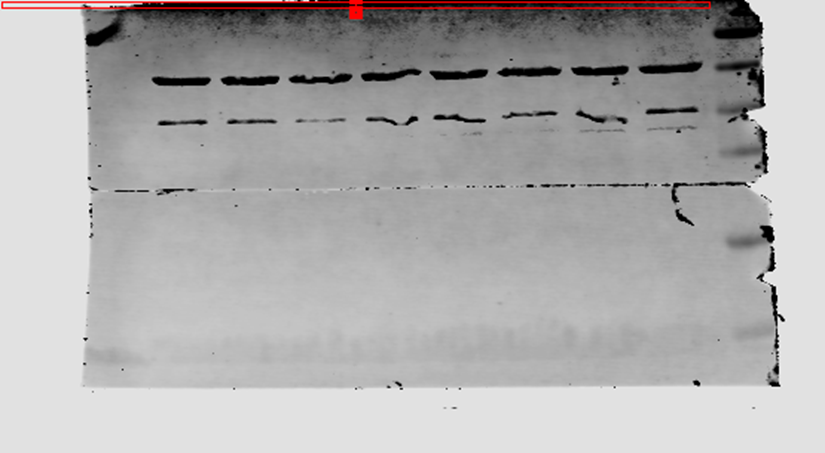


35 KD

25 KD

15 KD

55 KD

10 KD


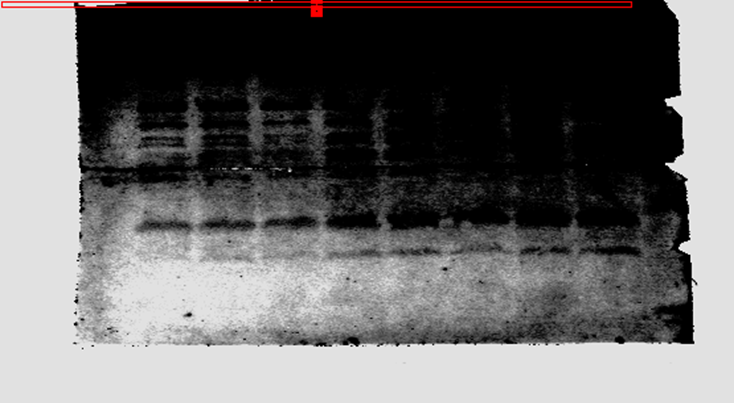


CXCL1

Figure 6A (p-EGFR)

120 60 30 0 120 60 30 0 120 60 30 0 min


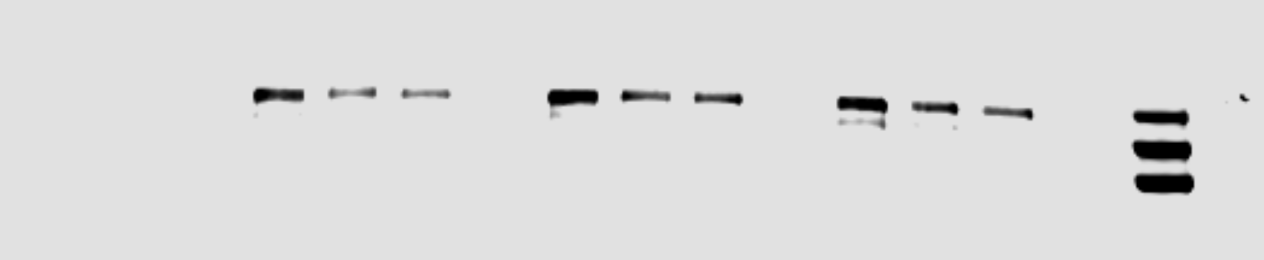


130KD

P-EGFR

100KD

170KD


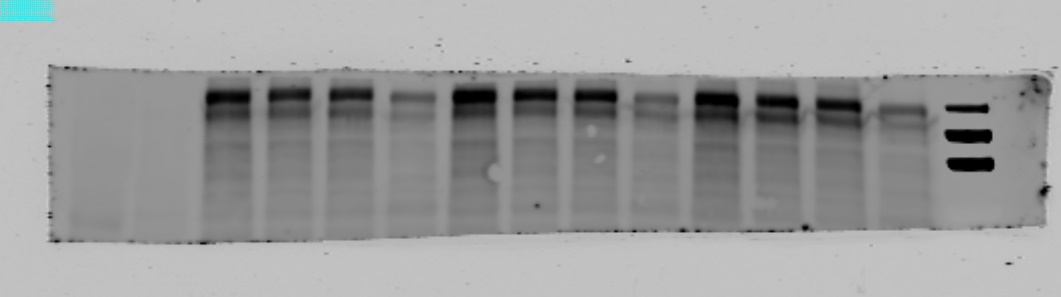


EGFR

55 KD


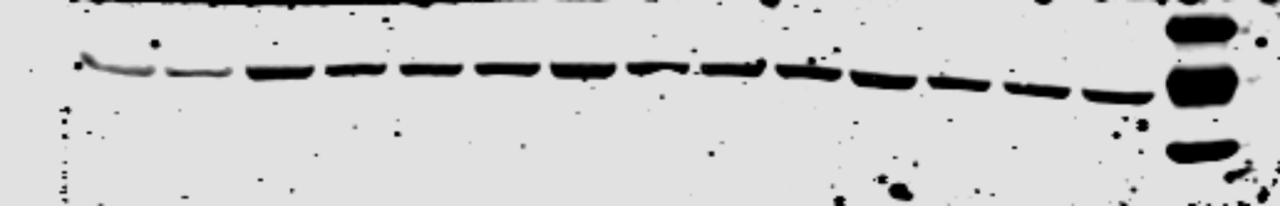


35 KD

40 KD

β-actin


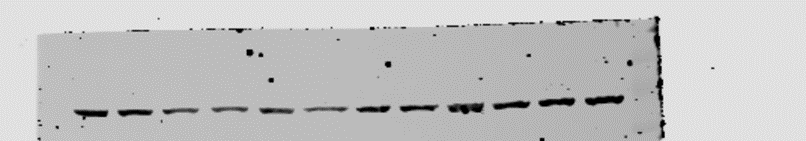


40 KD

55 KD

35 KD

β-actin

100KD


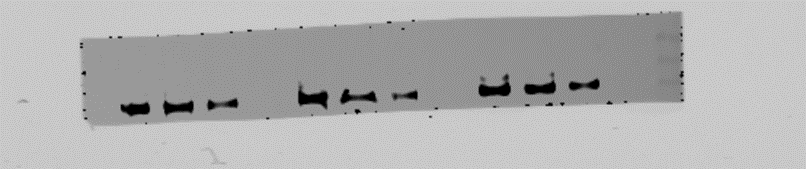


130KD

170KD

P-EGFR

Figure 6 D ( p-EGFR)


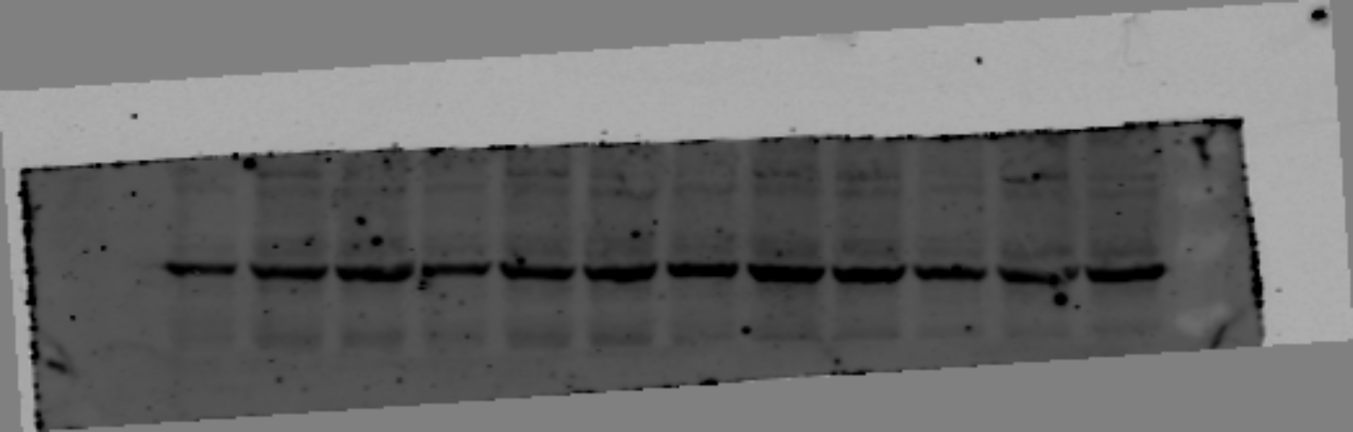


35 KD

40 KD

55 KD

β-actin


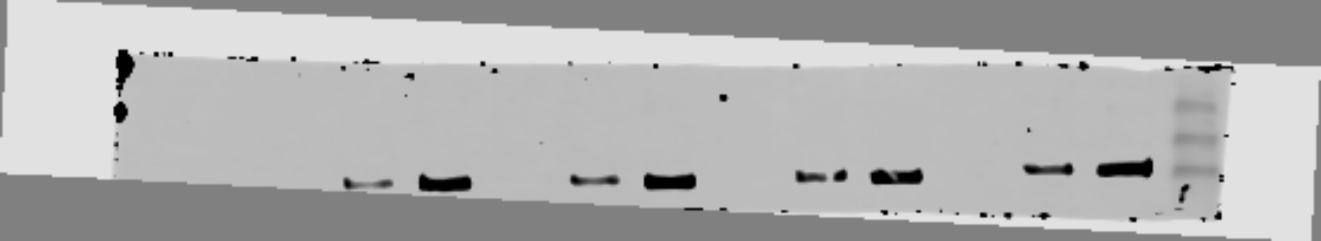


100 KD

130 KD

170 KD

P-EGFR


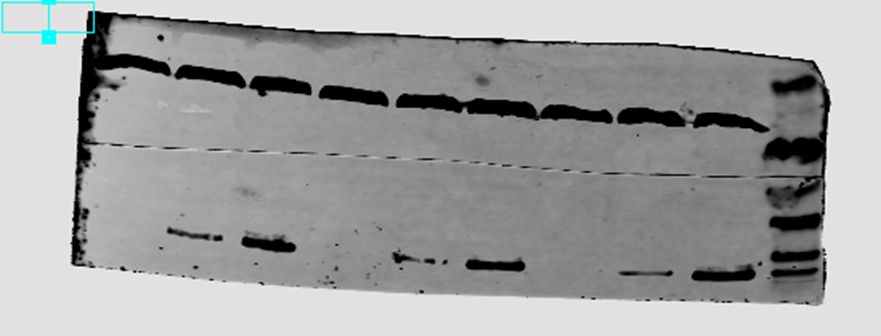


P-EGFR

40 KD

55 KD

70 KD

100 KD

130 KD

170 KD

Figure 6 D (p-P65/P65)


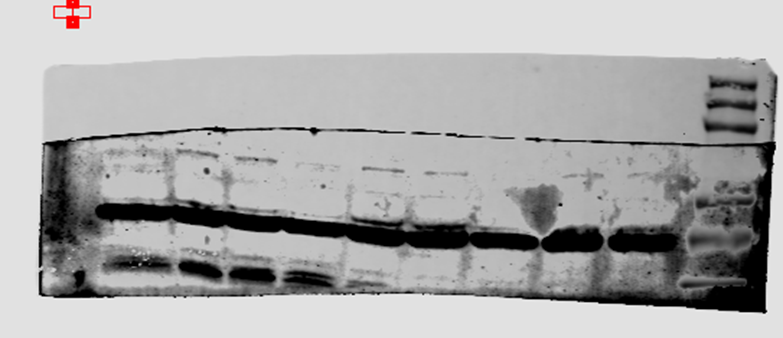


40 KD

130 KD

70 KD

55 KD

35 KD

100 KD

p-p65

35 KD

40 KD


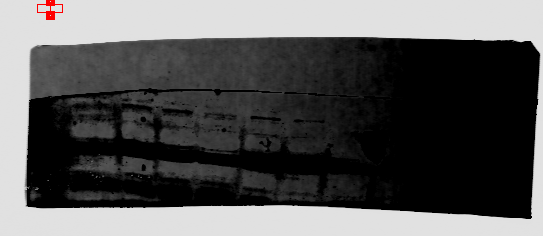


P65


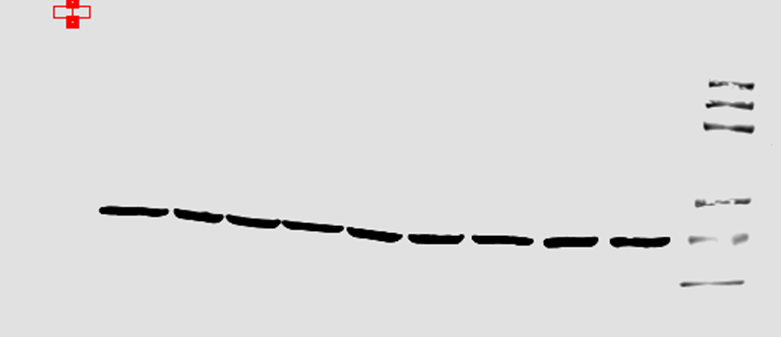


35 KD

40 KD

55 KD

70 KD

100 KD

130 KD

β-actin


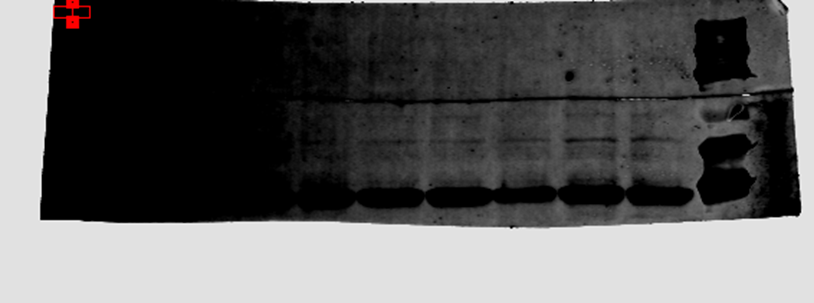


P-p65

55 KD

70 KD


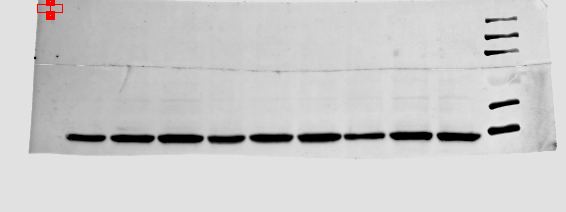

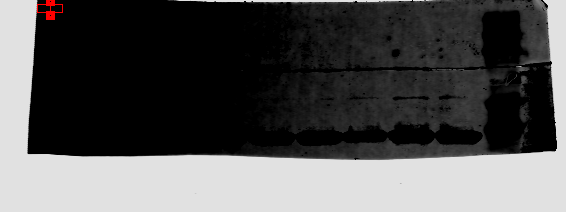


P65

40 KD

55 KD

70 KD

100 KD

170 KD

130 KD

β-actin


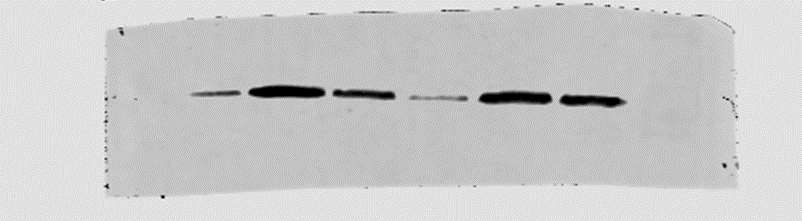


P-p65


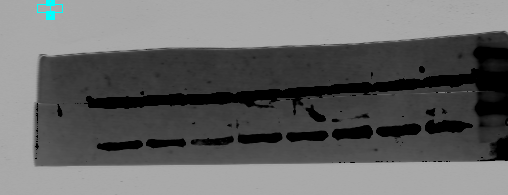


P65

55 KD

70 KD


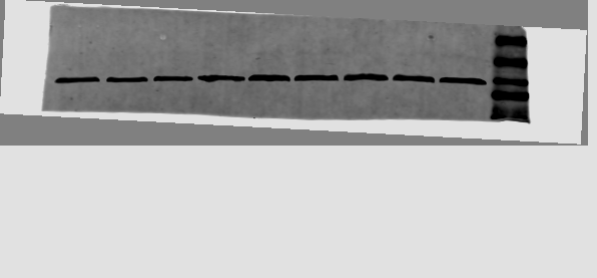


55 KD

40 KD

β-actin
